# Variability Assessment of Aromatic Rice Germplasm by Pheno-Genomic traits and Population Structure Analysis

**DOI:** 10.1038/s41598-018-28001-z

**Published:** 2018-07-02

**Authors:** M. Z. Islam, M. Khalequzzaman, M. K. Bashar, N. A. Ivy, M. A. K. Mian, B. R. Pittendrigh, M. M. Haque, M. P. Ali

**Affiliations:** 1 Genetic Resources and Seed Division, Bangladesh Rice Research Institute (BRRI), Gazipur, 1701 Bangladesh; 2 CIAT, HarvestPlus, Banani, Dhaka, 1213 Bangladesh; 3 Department of Genetics and Plant Breeding, Bangabandhu Sheikh Mujibur Rahman Agricultural University (BSMRAU), Gazipur, 1706 Bangladesh; 4 Department of Entomology, Michigan State University, East Lansing, MI USA; 5 Department of Agronomy, Bangabandhu Sheikh Mujibur Rahman Agricultural University (BSMRAU), Gazipur, 1706 Bangladesh; 6 Entomolgy Division, Bangladesh Rice Research Institute (BRRI), Gazipur, 1701 Bangladesh

## Abstract

While the pleasant scent of aromatic rice is making it more popular, with demand for aromatic rice expected to rise in future, varieties of this have low yield potential. Genetic diversity and population structure of aromatic germplasm provide valuable information for yield improvement which has potential market value and farm profit. Here, we show diversity and population structure of 113 rice germplasm based on phenotypic and genotypic traits. Phenotypic traits showed that considerable variation existed across the germplasm. Based on Shannon–Weaver index, the most variable phenotypic trait was lemma-palea color. Detecting 140 alleles, 11 were unique and suitable as a germplasm diagnostic tool. Phylogenetic cluster analysis using genotypic traits classified germplasm into three major groups. Moreover, model-based population structure analysis divided all germplasm into three groups, confirmed by principal component and neighbors joining tree analyses. An analysis of molecular variance (AMOVA) and pairwise FST test showed significant differentiation among all population pairs, ranging from 0.023 to 0.068, suggesting that all three groups differed. Significant correlation coefficient was detected between phenotypic and genotypic traits which could be valuable to select further improvement of germplasm. Findings from this study have the potential for future use in aromatic rice molecular breeding programs.

## Introduction

Rice is the staple food source for over half the world’s population. In Bangladesh, rice production occurs over an area of 11.4 million hectares (ha), generating 51.6 million tons of rice annually^1^, with 77% of the total cropped area being devoted to rice production, contributing more than 80% to the total food supply, and with rice providing 76% of the country’s countries caloric intact as well as 66% of its total required daily protein intake^2^. At present, rice alone constitutes about 93% of the total food grains produced annually in Bangladesh^3^.

Historically, thousands of local rice varieties have been cultivated across Bangladesh^4^ and local landraces, including aromatic ones, which have often been cultivated in less than favorable ecosystems that cover 12.16% of the total rice growing areas^1^. Some of these local varieties have desirable characteristics around aroma, better taste, and higher cooking quality, all of which potentiate value-added parameters to the rice both socially and economically. These aromatic rice germplasm constitute a small but important group of rice genotypes familiar in many countries of the world for their aroma or super-fine grain quality or both^5^.

To date, more than 8,000 varieties, landraces, cultivars, and wild-types of rice from indigenous and exotic sources are preserved in the Bangladesh Rice Research Institute (BRRI) genebank^6^, with more than 100 designated as aromatic varieties^7^. These aromatic germplasms are comprised of short and medium bold types with mild to strong aroma^8,9^.

In general, aromatic rice germplasm are tall-statured, possess a fewer number of panicles, have high stem weight, lower yields, and are susceptible to lodging and pest damage. Aromatic germplasm emits aroma (i.e., fragrance) due to the presence of a non-functional betaine aldehyde dehydrogenase 2 (BADH2), which is also responsible for low grain yield^10,11^. To date, no attempt has been made improve aromatic rice germplasm either geographically or genetically, and to the authors’ knowledge, no information on the genetic diversity of local aromatic rice germplasm has been published to date. Research into genetic diversity and correlation among the aromatic germplasm available in Bangladesh would not only play a vital role for hybridization to increase production, quality traits, stresses and tolerances, and in general provide information to help breeders identify pre-breeding materials^12^, but also would preserve information about this rice. Presently, the valuable genetic wealth of aromatic and fine-rice genotypes is being eroded because of their poor yield and the introduction of high-yielding varieties. Beyond more immediate commercial applications, it is essential that these valuable rice germplasms are collected, properly conserved, phenotypically and genotypically characterized, where possible genetically enhanced, and effectively documented within the context of their importance in intellectual property rights (IPR) regimes as well.

Genetic diversity in plants has long been assessed using morphological and physiological characters. Mahalanobis *D*^2^ statistics offer a powerful tool for determining clustering patterns as a way to establish relationships between genetic and geographical divergence as well as investigating the roles of different quantitative characters toward maximum divergence^13^. However, assessments based only on plant phenotypes are not a reliable measure of genetic difference given the influence of environmental conditions. The advent of PCR-based molecular marker technology, however, provides highly effective and reliable tools both for measuring genetic diversity in crop germplasm and evaluating evolutionary relationships within and between plant populations, varieties, and species^14^.

Across various molecular markers, simple sequence repeat (SSR) can serve as the marker for selection and affords some advantages over other markers^15–17^. They are abundant in eukaryotic organisms and often well-distributed throughout the genome^18,19^. Thus, SSRs are highly suitable for characterizing rice given their high reproducibility, simplicity, easy scoring ability, multi-allelic nature, hyper-variability, co-dominant inheritance, and genome-wide coverage^20^. SSRs are allele-specific and are co-dominant markers that have a high potential for identifying genetic diversity in an organism in a cost-effective manner^21,22^. To date, SSRs have been used for genetic diversity analysis, genetic characterization of genotypes, cultivar identification, marker-assisted selection breeding, and population structure assessment in multiple previously published rice genetic studies^23–31^.

## Results

### Phenotypic traits

**Figure 1:**
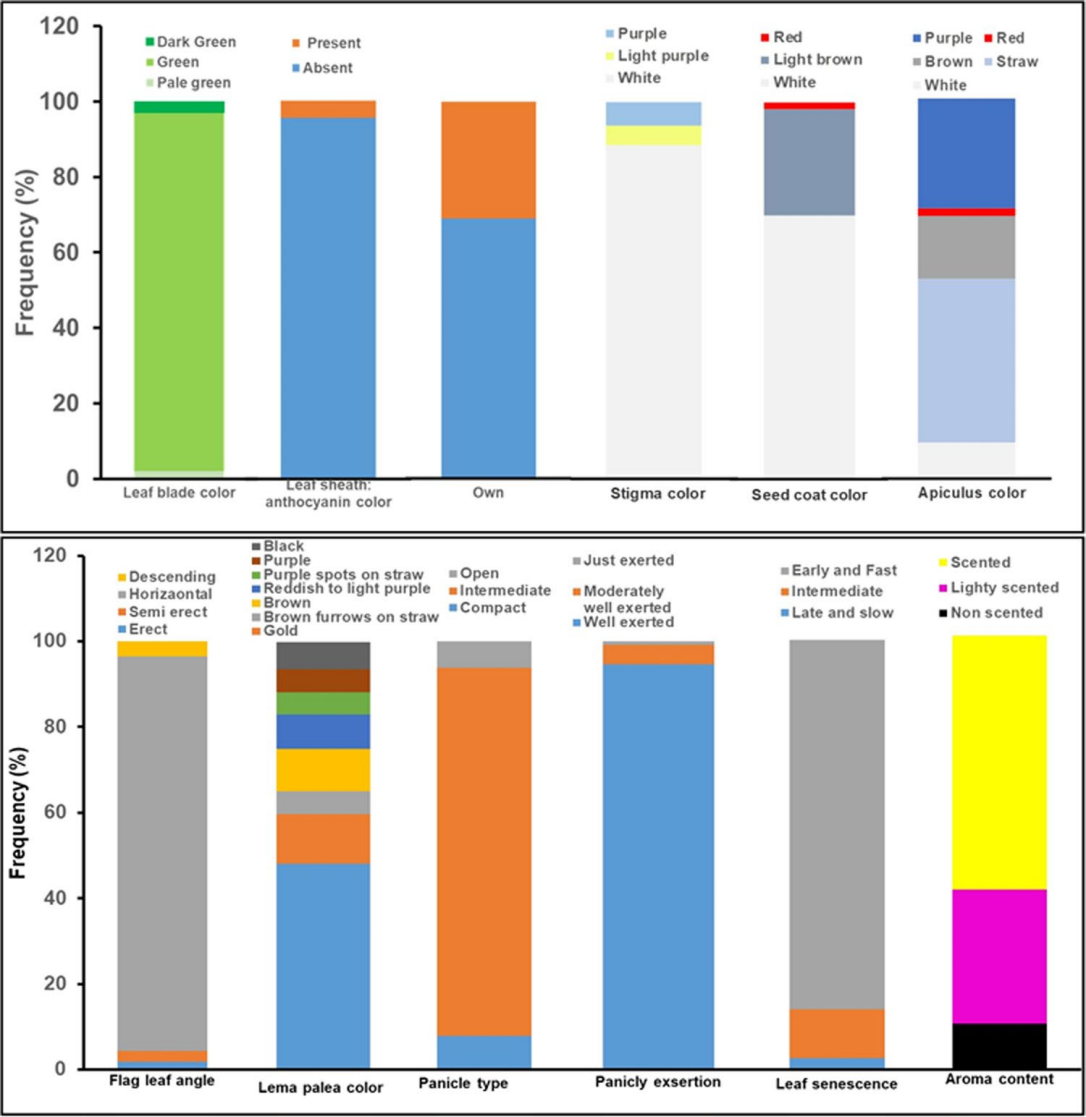
Frequency distribution of 113 aromatic rice germplasm based on 12 qualitative phenotypic traits.

We present, in Fig. 1, the frequency distribution for 113 aromatic germplasm for 12 qualitative phenotypic traits. Most of the germplasm (>96%) showed green leaf blade color. More than 95% germplasm showed anthocyanin leaf sheath color. Most germplasm (92%) had horizontal flag leaf, while 1.8%, 2.7% and 3.5% germplasm had erect, semi-erect, and descending flag leaf, respectively. Three types of panicle were observed, with 8%, 86% and 6.2% as compact, intermediate, and open-type, respectively. Panicles were found well exerted in 95% germplasm and 5% moderately well exerted. More than 69% germplasm were awnless, with 69.9% white seed coated. The most variable phenotypic qualitative traits of the tested germplasm were aroma content, apiculus color, and lemma-palea color (Fig. 1). More than 58% of the germplasm were well scented, while 31% germplasm were moderately scented. Several tested germplasm (11%), however, also seemed to be non-scented based on KOH extraction methods.

**Figure 2:**
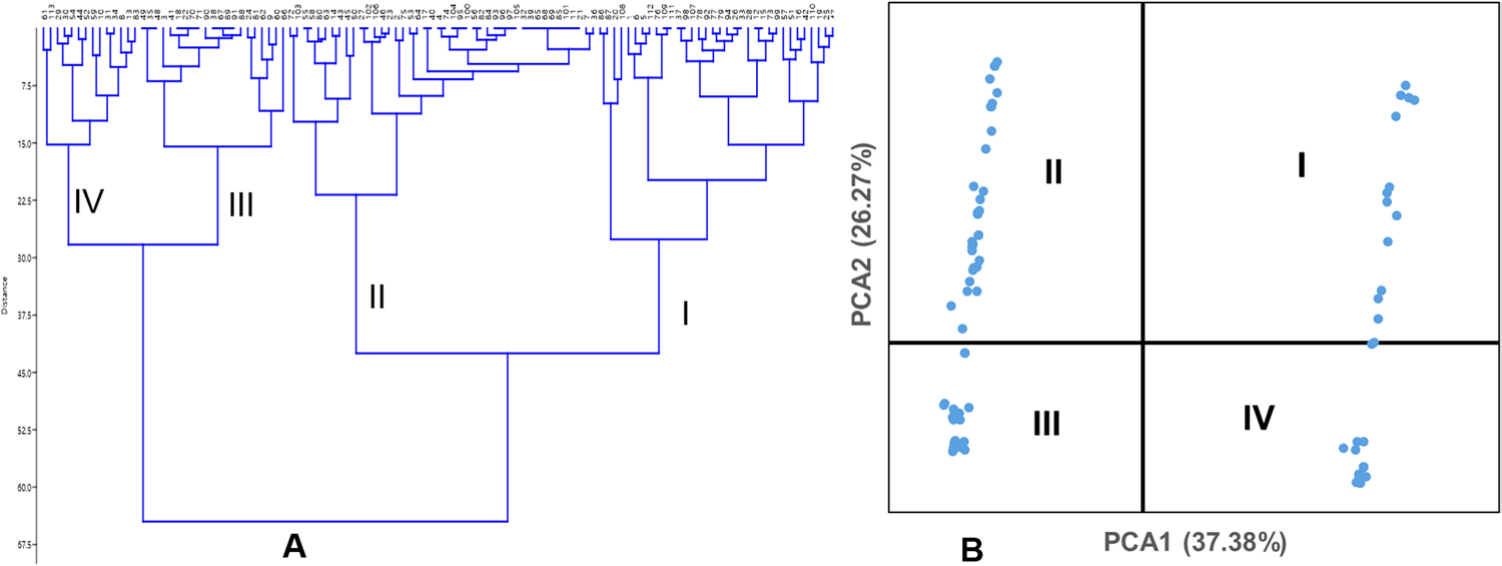
Dendogram and Principal Component Analysis (PCA) of tested germplasm based on 12 qualitative phenotypic traits.

**Figure 3:**
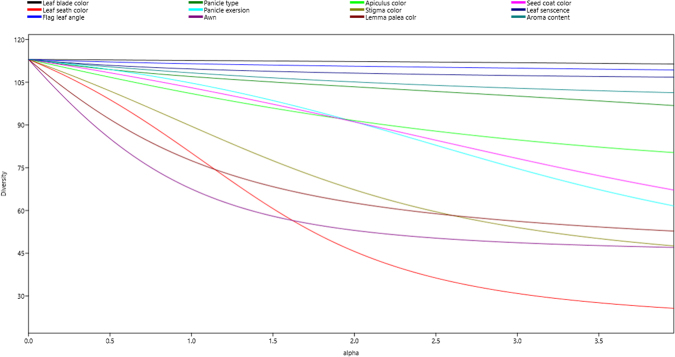
Diversity profile of tested germplasm based 12 qualitative phenotypic traits. This profile was developed using PAST software.

UPGMA-based dendrogram displayed major four groups for the 113 tested germplasm (Fig. 2A) based on 12 phenotypic qualitative traits. Group I, II, III, and IV consisted of 33, 45, 21, and 14 rice germplasm, respectively. PCA revealed that the first two components contributed 37.38% and 27.26% of the total variation, respectively. These two principal components were used to generate the group of the tested aromatic rice germplasm (Fig. 2B); Fig. 3 displays the resultant diversity profile. Increase in the *H*-based evenness value across the traits indicates the effective representativeness of the diversity available in the germplasm. Evenness varies from 0 to 1. Table 1 presents the Shannon–Weaver diversity index and evenness for the 12 phenotypic traits.

**Table 1:** Phenotypic variation of 113 aromatic rice germplasm based on 12 phenotypic traits. Both H′ and evenness were calculated using PAST software.

SL No.	Phenotypic trait	Descriptor states	Shannon diversity index (H′)	Evenness
1	Leaf senscence	3	0.476	0.9704
2	Flag leaf angle	5	0.362	0.9865
3	Panicle type	3	0.505	0.9467
4	Apiculus color	5	1.319	0.8938
5	Aroma content	3	0.915	0.9581
6	Leaf blade color	3	0.139	0.9968
7	Lemma palea color	9	1.711	0.6856
8	Awn	2	0.619	0.5977
9	Seed coat color	3	0.678	0.912
10	Stigma color	3	0.436	0.7924
11	Panicle exersion	3	0.231	0.9265
12	Leaf sheath color	2	0.181	0.7095

Generally, the Shannon–Weaver diversity index value represents the degree of diversity prevailing among the tested samples. Higher value indicates higher diversity and vice versa. In this study, the Shannon–Weaver index values ranged from 0.14 for leaf blade color to 1.71 for lemma-palea color, the latter showing considerable variation.

### SSR polymorphism among aromatic rice germplasm

Overall, 52 well-spread microsatellite (SSR) markers covering all 12 chromosomes were used to characterize and assess genetic diversity among 113 aromatic rice germplasm. Only 45 markers, however, showed clear and consistent polymorphic banding patterns and amplification of each genotype, indicating that these microsatellites were suitable for genetic diversity analysis. The remaining seven markers produced monomorphic bands revealing one allele at each locus in all the germplasm (not discussed here) and hence were not useful for this study.

**Figure 4:**
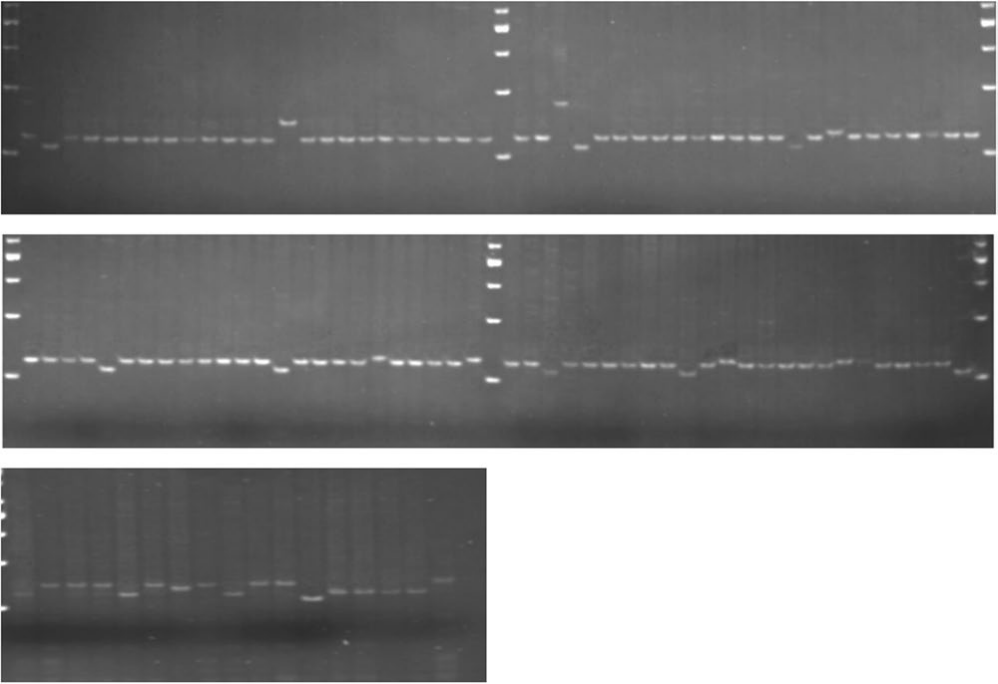
Banding pattern by marker RM447 for tested germplasm (gel picture). Upper and middle parts of the figure were the one gel picture. Both upper and middle figures represent banding pattern of 96 germplasm. We divided two parts and showed here for recording band length precisely. This small part provide accurate band length in measuring software. However, lower part of the figure is another gel which represent banding pattern of 17 rice germplasm.

The gel picture for banding of the 113 aromatic rice germplasm with the RM447 marker is shown in Fig. 4. These loci were applied to discriminate morphologically uniform and non-uniform germplasm. The number of alleles, allele size, highest frequency allele, rare alleles, unique alleles, and polymorphism information content (PIC) detected among 113 germplasm are presented in Table 2.

**Table 2:** Number of alleles, allele size, major allele frequency and polymorphism information content (PIC) observed among 113 test germplasm for 45 SSR markers.

Marker	Chro. No.	Position (cM)	Motif*	Allele No.	Rare alleles	Unique alleles	Size range (bp)	Highest frequency allele		PIC Value
								Size (bp)	Freq(%)	
RM5	1	94.9	(GA)14	3	0	0	107–123	123	55.91	0.5225
RM495	1	2.8	(CTG)7	3	0	1	138–158	158	49.48	0.3901
RM431	1	178.3	(AG)16	3	1	0	230–246	230	53.64	0.4702
RM237	1	115.2	(CT)18	4	2	0	69–134	128	48.24	0.6052
RM312	1	71.6	(ATTT)4(GT)9	3	1	1	100–108	104	90.27	0.1644
RM283	1	31.4	(GA)18	4	0	0	151–168	155	37.21	0.6446
RM452	2	58.4	(GTC)9	2	1	0	192–202	192	96.46	0.0660
RM6	2	154.7	(AG)16	3	1	1	146–166	146	92.86	0.1269
RM322	2	49.7	(CAT)7	2	1	0	110–115	115	93.41	0.1156
RM489	3	29.2	(ATA)8	4	2	0	194–322	271	71.95	0.4100
RM338	3	108.4	(CTT)6	2	1	0	183–188	188	94.59	0.0970
OSR13	3	53.1	(GA)n	4	1	1	100–114	104	68.57	0.3886
RM514	3	216.4	(AC)12	2	1	0	260–275	260	92.08	0.1352
RM307	4	0	(AT)14(GT)21	3	2	0	134–165	134	96.36	0.0695
RM537	4	8.5	(CCG)9	2	1	0	231–240	231	90.91	0.1516
RM551	4	8.5	(AG)18	3	1	0	186–216	193	80.231	0.2839
**RM178**	**5**	**118.8**	**(GA)5(AG)8**	**2**	**1**	**0**	**120–127**	**120**	**97.30**	**0.0512**
RM413	5	26.7	(AG)11	3	1	0	69–90	69	90.40	0.1703
RM510	6	20.8	(GA)15	2	0	0	108–115	108	87.06	0.2002
RM454	6	99.3	(GCT)8	3	2	0	272–312	272	87.16	0.2179
RM170	6	2.2–7.4	(CCT)7	4	2	0	105–121	121	50.00	0.5040
RM190	6	7.4	(CT)11	3	0	0	105–125	120	73.64	0.3840
RM253	6	37	(GA)25	5	1	0	120–148	136	57.80	0.4995
RM314	6	33.6	(GT)8(CG)3(GT)5	3	2	0	111–123	116	42.20	0.5810
RM455	7	65.7	(TTCT)5	2	1	0	131–135	131	97.20	0.0517
RM118	7	96.9	(GA)8	2	1	0	156–161	156	91.89	0.1379
RM125	7	24.8	(GCT)8	3	2	0	124–135	128	85.71	0.2373
RM10	7	63.5	(GA)15	2	1	0	169–164	169	93.69	0.1112
RM408	8	0–1.1	(CT)13	2	0	0	123–129	129	86.79	0.2030
RM25	8	52.2	(GA)18	4	3	0	131–146	131	75.49	0.3865
RM44	8	60.9	(GA)16	3	0	1	95–111	111	83.16	0.2488
RM284	8	83.7	(GA)8	5	3	1	72–102	72	89.32	0.1926
RM447	8	124.6	(CTT)8	4	2	1	104–134	112	82.30	0.2890
RM223	8	80.5	(CT)25	5	2	1	142–164	150	55.36	0.5127
RM342	8	78.4	(CAT)12	4	1	0	116–148	122	35.40	0.6655
RM515	8	80.5	(GA)11	5	2	0	212–261	244	68.47	0.4707
RM316	9	1.8	(GT)8-(TG)9(TTTG)4 (TG)4	3	0	0	199–214	207	39.42	0.5721
RM215	9	99.4	(CT)16	3	2	0	148–160	153	88.46	0.1985
RM271	10	59.4	(GA)15	3	1	1	92–102	96	95.83	0.0785
RM287	11	68.6	(GA)21	3	1	1	95–115	105	95.65	0.0817
RM536	11	55.1	(CT)16	3	1	1	230–245	245	92.38	0.1343
**RM144**	**11**	**123.2**	**(ATT)11**	**5**	**2**	**0**	**227–290**	**240**	**52.18**	**0.6707**
RM19	12	20.9	(ATC)10	3	2	0	220–245	220	94.39	0.1041
RM20	12	0	(ATT)14	2	0	0	208–225	208	84.00	0.2327
RM277	12	57.2	(GA)11	2	0	0	120–125	120	78.72	0.2789
Total	—	—	—	140	52	11	—	—	3463.541	13.1078
Mean	—	—	—	3.11	1.15	0.24	—	—	76.96	0.2912

Note:Major allele is described as the allele with the highest frequency. Rare alleles are described as alleles with a frequency less than 5%.

Overall, 140 alleles were identified from the 113 tested germplasm with an average 3.11 alleles. The highest PIC value (0.67) was found for the marker RM114, with the lowest (0.051) observed for marker RM178, with a mean of 0.29. The most frequent major allele frequency (0.97) was found for the marker RM455 and the lowest (0.37) for marker RM283, with a mean of 0.77.

Rare alleles are described as alleles with a frequency less than 5%. In general, markers detecting a greater number of alleles per locus detected more rare alleles. Fifty-two (52) rare alleles were identified at 45 SSR markers with an average of 1.15 alleles per locus (Table 2). The highest number of rare alleles was observed at the RM25 and RM284 loci (3 alleles), followed several markers (Table 2). Eleven unique alleles were also detected using 11 SSR markers specific to a given germplasm (Table 3); for instance, the popular rice variety “BRRI dhan50” was uniquely identified using RM6, “Sakkorkhora” with RM44, and “Bashful” with RM536.

**Table 3:** The unique alleles found in 11 germplasm out of 113 germplasm were tested using 52 SSR markers.

SL No.	Marker	Chromosome	Unique allele (bp)	Name of genotypes
1	RM495	01	138	Sugandhi dhan
2	RM312	01	108	Chini kanai
3	RM6	02	166	BRRI dhan50
4	RM44	08	95	Sakkorkhora
5	RM284	08	86	Elai
6	RM271	10	92	Khazar
7	RM536	11	230	Bashful
8	RM287	11	95	Begunbichi
9	RM447	08	134	Begunbichi
10	OSR13	03	62	Straw TAPL-554
11	RM223	08	142	Straw TAPL-554

### Genetic relationship among the germplasm

**Figure 5:**
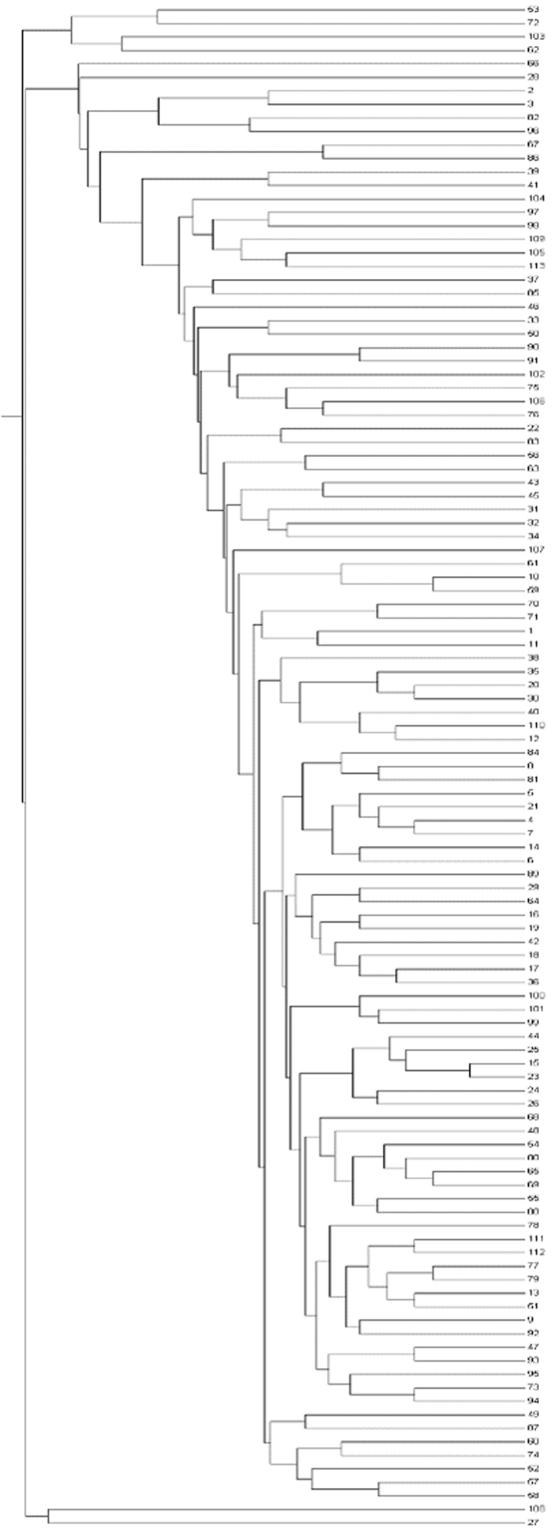
Dendogram of tested germplasm. Dendogram was generated using MEGA software.

An unrooted neighbor-joining tree, which showed the genetic relationships among the 113 tested germplasms, was constructed based on the alleles detected by 45 SSR markers. The genetic distance-based results stated in the unrooted neighbor-joining tree revealed three groups in the 113 tested germplasm (Fig. 5). We also constructed an UPGMA-based dendrogram to generate the genetic distance among the groups. Analysis of the pooled SSR marker data had an average similarity co-efficient of 0.44. The analysis also showed genetic variation among the aromatic rice germplasm tested, with similarity coefficient values ranging from 0.39 to 0.93, demonstrating a moderate degree of genetic diversity among the germplasm used in this study. Moreover, the three dimensional (3D) graphical view of the principal component analysis (PCA) showed the spatial distribution of the 113 tested germplasm along the three principal axes, with most closely associated near the centroid in the 3D graph (data not shown).

### Spearman rank correlation test between D^2^ and SSR rankings

Spearman rank correlation was conducted to compare the morphological and molecular data for distinguishing germplasm. The 6328 genetic distances between germplasm measured through D^2^ and SSR analysis were ranked separately (Supplementary information, Table S1) and assessed by Spearman’s rank correlation formula, with ${r}_{s}$ = 0.276 and significant at t = 3.41 (p = 0.03) with two degrees of freedom, indicating that a statistical association between phenotypic and genotypic traits existed. The significant (p = 0.03) correlation coefficient between the D^2^ analysis and the ranking of SSR markers, based on Nei distance, reflected that both of these two techniques were very effective for grouping the germplasm. Supplementary information (Table S1) presents the cumulative Nei distance ranking and D^2^ genetic distance ranking position for all tested germplasm. By Nei distance ranking, StrawTAPL-500 and Desi Katari stood first and last rank, respectively; by D^2^ distance ranking, however, Kalobakri and Tilkapur ranked first and last, respectively. These results indicated that a statistical association between groups, phenotypic and genotypic traits existed.

### Population structure model based approach

**Figure 6:**
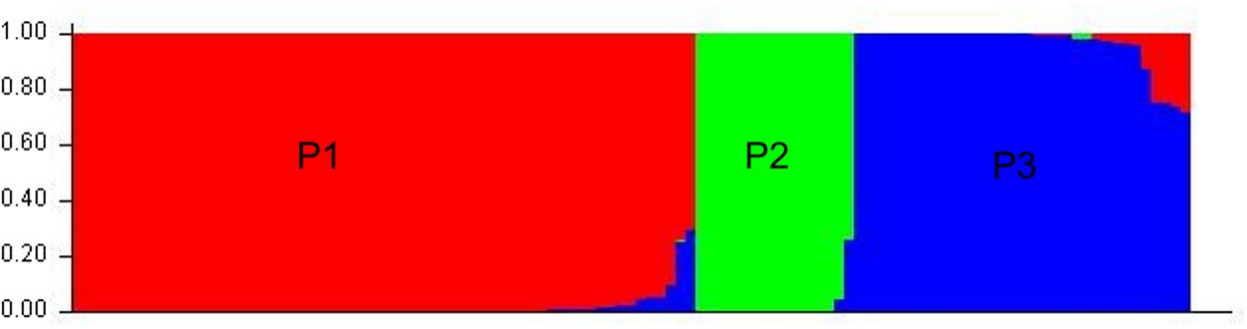
Assignment of tested germplasm to population P1, P2, and P3. The population structure was determined using STRUCTURE 2.3.4 software.

A model without admixture was carried out varying K from 1 to 15 with five iterations using all 113 germplasm and 45 polymorphic markers for maximum likelihood and delta K (nK) values (data not shown). At K = 3, all germplasm stratified into three populations (P1, P2, P3), representing 56%, 14% and 30% of germplasm used in structure analysis respectively; Fig. 6 presents the inferred population structure. Moreover, based on the membership fractions, germplasm under these three population were classified as pure or an admixture: P1 showed 58 pure (90.6%) and 6 admixed (9.4%) individuals, P2 had 14 pure (87.5%) and 2 (12.5%) admixed individuals, and P3 had 29 pure (87.9%) and 4 (12.1%) admixed individuals.

**Figure 7:**
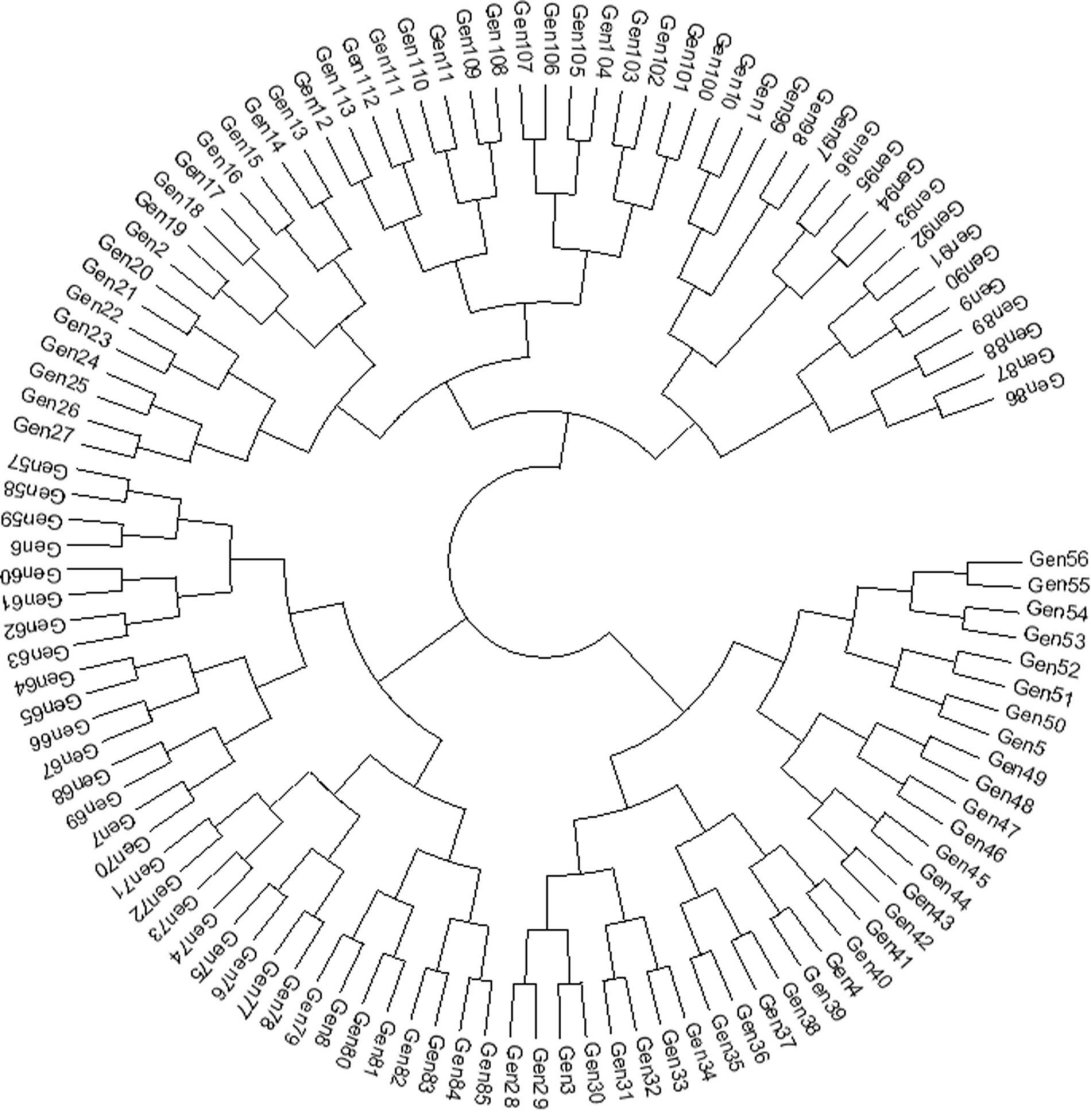
Neighbour-joining tree of tested germplasm by model-based approach derived population.

**Figure 8:**
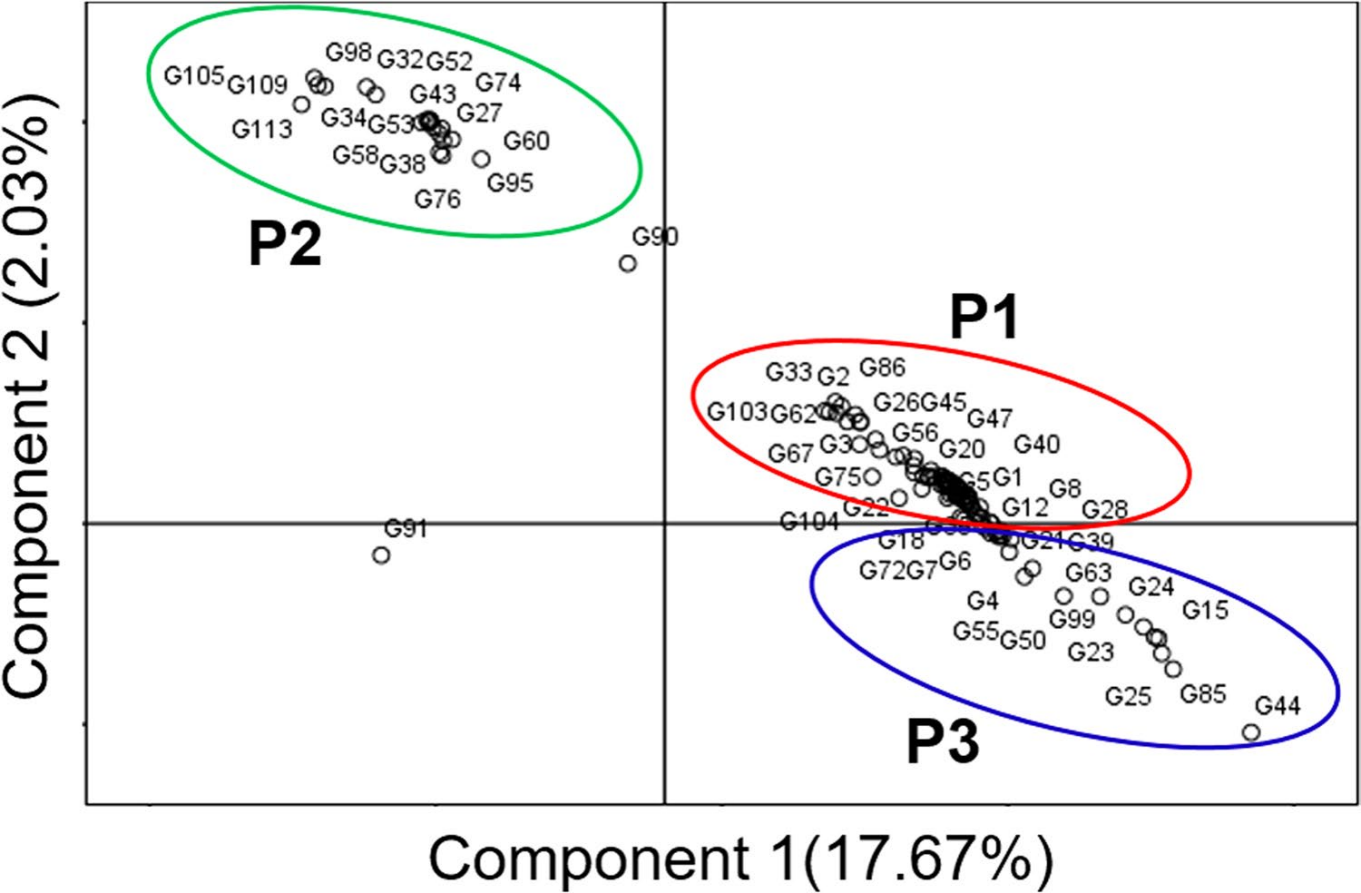
Principal Component Analysis (PCA) of tested germplasm by model-based approach derived population.

FST statistics tested genetic variation among the three population with values of 0.39 0.11 and 0.28 for P1, P2, and P3, respectively, with an average 0.26, indicating a moderate population structure. Thus, the most structured population was P1, followed by P3, and then the P2 population. The specific FST values (not the pair-wise FST values between populations) for the three populations were calculated using STRUCTURE. The expected heterozygosity or averaged distances between individuals, in same cluster, were 0.23, 0.47, and 0.30, respectively. The largest genetic (net nucleotide) distance (0.12) was observed between P1 and P2, while, the genetic distances between P1 vs P3 and P2 vs P3 were 0.023 and 0.09, respectively. The neighbor-joining (NJ) tree and principal component analysis (PCA) based on population derived from the structure analysis were conducted. Both the NJ tree and PCA analyses further confirmed the STRUCTURE results. The tree model-based groups (P1–P3) were clearly separated in the NJ tree (Fig. 7). In the PCA (Fig. 8), the first two eigenvectors clearly separated the overall germplasm into three major groups, which was similar to what was observed in STRUCTURE analysis and NJ tree.

### Analysis of molecular variance

The three populations generated from the above structural analysis were also subjected to analysis of molecular variance (AMOVA) to estimate the percentage of variation between populations and within populations. In the total genetic variance between populations, based on structure, we observed that 6% was attributed to the populations and the remaining 93% was explained by individual differences within populations (Table 4). Approximately 1% of the total observed genetic variance could be explained by differences at the level of the individual. Pairwise FST values showed significant differentiation among all the pairs of populations, ranging from 0.02 to 0.07, suggesting that all of the three groups were different from each other. The P1 and P3 populations had the greatest level of differentiation from each other, as determined by the FST estimate (Supplementary information, Table S2).

**Table 4:** Analysis of molecular variance (AMOVA) of 113 aromatic rice germplasm available in Bangladesh.

Source of variation	df	SS	MS	CV	Variation (%)	P value
Among Population	2	117.611	58.806	0.590	6%	0.001
Among Individual	110	2203.628	20.033	9.952	93%	0.001
Within Individual	113	14.500	0.128	0.128	1%	0.001
Total	225	2335.739		10.671	100%	

Notes: df, Degrees of freedom, SS, Sum of squares, CV, Variance component estimates, % Total, percentage of total variation.

In general, the results from AMOVA and FST analysis were in agreement with the observations obtained through (i) the phylogenetic tree-based, (ii) similarity coefficient distribution, and (iii) structure analysis, confirming the presence of both a statistically moderate genetic diversity and a high level of population structure.

## Discussion

Exploitation of genetic diversity, present in a crop species, can improve the target traits of that crop for the crop breeder, farmers, and ultimately consumers^17^. While cultivated varieties of aromatic rice in Bangladesh developed as a result of farmers’ and scientists’ selection from within the existing and available genetic diversity in a diversity of environments, one can argue that modern breeding over the past two centuries, in some cases, has resulted in the release or maintenance of varieties that are considered uniform, less stable, and better adapted to controlled and limited environmental conditions^32^. This makes narrow genetic background varieties popular among farmers, even as these improved varieties may, in some cases, be more susceptible to biotic and abiotic stresses.

In Bangladesh (as elsewhere), aromatic rice improvement requires (i) the identification of highly diverse germplasm, (ii) highly polymorphic molecular markers that, (iii) in turn, can be effectively utilized for the mapping of genes/QTLs for economically important traits and (iv) where a subset of these markers can be used in molecular breeding programs towards the development of improved varieties. Thus, steps towards understanding varietal characteristics of aromatic rice has the potential to play a vital role in future national and international breeding programs, especially where aromatic traits may be desirable for consumers. This research is a first step in a broader initiative to characterize the genetic base and improve the aromatic rice germplasm commonly grown in Bangladesh and conserved in the BRRI Genebank.

We used 12 qualitative phenotypic traits to classify the 113 germplasm into four groups. These phenotypic traits can be used for rapid identification of germplasm bank materials. Phenotypic traits of crop germplasm have been widely used for diversity analysis^33,34^. Because qualitative and quantitative phenotypic traits can impact the genetic diversity of rice germplasm^35^, they can be used for evaluating that genetic diversity^36,37^. Specifically, the degree of diversity can be evaluated using Shannon-Weaver diversity index value (*H*). We observed the highest *H* in lemma palea color, but the phenotypic traits used for diversity analysis had considerable variation (Table 1).

The advent of molecular markers, and their use for the identification of diverse germplasm is highly advantageous over previous approaches^38^. Among different types of molecular markers, SSRs have been widely utilized in the study of genetic diversity analysis, genotypic grouping, and population structure analysis for numerous crop species, including aromatic rice^15,17,39–41^. In the present study, selected SSR markers were determined to be suitable for a genetic diversity analysis of aromatic rice germplasm available in Bangladesh. The markers produced unique, rare and major allelic profiles for the 113 aromatic rice germplasm, with PIC values ranging from 0.05 to 0.67, with an average of 0.29; the genetic diversity observed in this study falls within the ranges found in several earlier studies^15,17,42^.

Previous studies detected different numbers of allele per locus: e.g., three alleles per locus and an average PIC of 0.41^17^, 4.8 alleles per locus and an average PIC value of 0.50^42^, three alleles per locus with an average PIC value of 0.41^31^, and 8.03 alleles per locus with an average PIC value of 0.62^15^. Similarly, an average PIC value of 0.44 was observed among 43 Thai and 57 IRRI germplasm of rice^43^. In another study, conducted by Thomson and co-workers, they observed an average PIC value of 0.45 among the 183 Indonesian rice landraces collected from across the islands of Borneo^44^. Shah and co-workers reported a slightly lower level of genetic diversity, averaging 2.75 alleles per locus as well as an average PIC value of 0.38 amongst the 40 rice accessions they tested from Pakistan^22^. Additionally, Singh and co-workers observed a lower SSR diversity in a study with 36 polymorphic HvSSRs, whereby they detected 2.22 alleles per locus with an average PIC value of 0.25 from a total of 375 Indian rice varieties which were collected from a diversity of regions across India^21^.

The PIC value of a marker is the probability of the marker allele that can be deduced in the progeny and is a good measure of a marker’s usefulness for linkage analysis^45^. In general, higher PIC values were observed for SSRs having higher number of alleles while lower PIC value indicated that the genotypes under study are closely related; higher PIC values, conversely, indicate higher diversity in the germplasm being tested and such germplasm is better suitability for the development of new varieties. In our study, the primer RM 144 had the highest PIC value (0.67) and the highest number of alleles (5), indicating that it detected the highest level of polymorphism and the best marker for characterizing the aromatic rice germplasm. Markers RM342, RM 283, RM237, and RM 314 were also useful for molecular characterization of germplasm, though to a lesser extent.

Some of the SSR markers also generated germplasm-specific bands that can be used as molecular identity data for specific germplasm. For example, marker RM144 amplified all germplasm and showed specific fragments (Fig. 4). Overall, 52 rare alleles, with a frequency less than 5%, were identified across the tested rice germplasm, or an average 1.15 rare alleles per SSR marker, which is lower than in other similar reports^46–48^.

Identification of unique alleles has a great importance both for identifying specific genotypes but also for breeding^49,50^. In this study, nine unique alleles were identified by SSR markers (Table 3), and each germplasm showed unique alleles for at least one microsatellite locus. However, the number of unique alleles per locus varied from one to two^49^. Seven aromatic rice germplasm—“Sugandhi dhan,” “Chini Kanai,” “BRRI dhan50,” “Khazar,” “Bashful,” “Sakkorkhora,” and “Elai”—each amplified one unique allele (Table 2). Additionally, both “Begunbichi” and “Straw TAPL-554” showed two unique alleles. Generally, the higher number of unique alleles in a germplasm indicates its potential as a reservoir of novel alleles for crop improvement. The findings here are similar to other research. For example, Saini *et al*.^51^. identified 58 unique alleles among rice using SSR marks, with all unique alleles found at 25 of 30 SSR loci. Similarly, unique alleles are detectable in both cultivated and wild rices^52^.

Diversity analysis relies on genotypic, phenotypic and geographic information of a crop species. Correlation coefficient analysis provides useful knowledge in terms of quantitative, qualitative characteristics issuing a credibility and important attributes about a species^53^. In this study, we determined correlation between phenotypic and genotypic traits of the tested germplasm which showed significant statistical relationship between two groups of data. This relationship is highly desirable and has significant value and used for selection because phenotypic traits are dependent on genotypic traits^54^.

Aromatic rice germplasm is composed of small-, medium-, and long-grain types with mild to strong aroma^8,9^. Based on conventional taxonomy, Bangladeshi aromatic rice has been classified as *indica*. Subsequent studies, which have been based on SSR and isozyme markers, have demonstrated that most of the aromatic rice cultivars from the Indian sub-continent, which includes Basmati (scented) types, have been characterized as a genetically distinct cluster^17,55–58^. Based on SSR marker analysis, Roy *et al*.^15^ reported three major groups from a set of 107 Indian aromatic rice accessions. In the present study, population structure analysis also revealed three populations (P1–P3) with a majority of germplasm in P1. This grouping agrees with genetic distance based clustering and PCA (Figs 7 and 8). While, to the authors’ knowledge, this is the first genetic structure study for aromatic Bangladeshi germplasm, analysis of 141 aromatic Indian rice genotypes demonstrated five sub-populations^17^. Choudhury *et al*.^25^ found two clusters within 24 indigenous and improved rice varieties in northeast India, while Das *et al*.^26^ found four groups among a set of 26 rice cultivars.

In this study, genetic diversity among the tested germplasm was also evaluated by a model-based structure using the SSR genotypic data. The genetic architecture of diverse germplasm can be estimated by determining the STRUCTURE of the population using molecular markers such as, but not limited to, SSRs or SNPs^16,21,30,40,59,60^. In STRUCTURE, LnP(D) denotes the highest optimal number of subsets (K)^61–63^ for an optimal number of divisions^64^. In this study, at K = 3, all 113 germplasm divided into three population, with 63 in P1, 17 in P2, and 33 in P3 (Fig. 6), indicating genetic differentiation in the overall germplasm.

Out of the total tested, ten were non-scented but were distributed in all three population: four each in P1 and P2 and two in P3. Similarly, the majority of scented germplasm were observed in P1. Four of five high-yield varieties developed by BRRI—“BR5,” “BRRI dhan34,” “BRRI dhan37,” and “BRRI dhan38”—were distributed in P1; “BRRI dhan50” was in P3. In Bangladesh, germplasm are typically classed by grain shape and size^65^. Most of the long- and slender-type germplasm were found in P2 population. Other grain types, e.g., short bold and short-medium germplasm, were found in P1 and P3.

## Conclusion

Phenotypic traits and SSR marker based molecular characterization of 113 aromatic Bangladeshi germplasm confirmed genetic variation among the germplasm. The phenotypic traits classified the tested germplasm into major four groups. However, the population structure analysis allowed us to identify three major groups within these germplasm and this that generally agrees with the farmers’ classification of the germplasm. Future population genetics-based studies that include extensive collections of rice genetic resources from all of the districts of Bangladesh would help in exploiting this rice gene pool more effectively for rice improvement program. Bangladesh has 64 districts where rice grows every year and usually farmers cultivate some local germplasm with high yielding varieties. In this study we only collected germplasm from 22 districts, remaining all other districts may have many other germplasm that will provide wide diversity and can be used in future rice breeding programme for improvement of aromatic rice in Bangladesh as well global if they will be collected and preserved in geebank.

In both domestic and international markets, aromatic rice commands a premium price, often two to three times higher than traditional rice cultivars due to consumer quality preferences. Some of the traditional aromatic rice varieties of Bangladesh—including “Kataribhog,” “Chini Sagar,” “Kalobhog,” “Chini Atob,” “Noyonmoni,” “Chinnigura,” “Gopalbhog,” “Tulsimoni,” “Jirabuti,” “Khirshaboti,” “Rajbut,” and “Kalijira”—are cultivated throughout the country for traditional and consumer-preference reasons. Low yield of these high-value rice, however, limit their market potential. Better understanding the genetic diversity preserved in this aromatic rice gene pool will facilitate proper maintenance, conservation, and utilization of this valuable resource.

## Materials and Methods

### Materials

In this study, we used 45 polymorphic SSR markers to analyze the genetic diversity of 113 aromatic rice germplasm representing landraces, fine rice genotypes, elite cultivars, and exotic genotypes preserved in BRRI genebank in Gazipur, Bangladesh. Names for the 113 aromatic rice germplasm along with quantitative phenotypic traits have been previously described^7^. Seeds from BRRI are publicly available for research purposes upon request with a materials transfer agreement.

For our analysis, 52 SSR markers were used from the ‘Gramene’ marker database^66^. Out of these 52 SSR markers, 45 were polymorphic, in terms of their bands, among the rice varieties, while seven were monomorphic; the marker names and their respective sequences are presented in Supplementary information (Table S3). The 45 polymorphic markers selected for analysis were distributed across the 12 chromosomes, whereby three were linked to aromatic traits, four were related to cooking and eating quality traits, 31 were listed in the panel of 50 standard SSR markers used for diversity analysis^66^ and the remainder of the SSRs were selected randomly.

### Analysis of phenotypic traits

The seed of each tested germplasm was taken from BRRI gene bank and grown in the BRRI research field following a rice production technology previously described by Islam *et al*.^7^. Data on 28 qualitative phenotypic traits were recorded. Ten randomly selected plants from each germplasm were used for recording the respective phenotypic traits data. Each phenotypic trait with their descriptors, according to BRRI^67^, are shown in Table 1. All other quantitative phenotypic traits have been previously reported^7^. The most important 12 qualitative phenotypic traits selected as follows: leaf blade color, leaf sheath color, flag leaf angle, lemma-palea color, seed coat color, stigma color, awn in the spikelet, apiculus color, leaf senescence, aroma content, panicle type, and panicle exertion. We used all 12 traits in the following statistical analyses. Shannon–Weaver diversity index (*H*), evenness, and diversity profile, as well as clustering and PCA analyses of 12 phenotypic traits, were determined using PAST (PAleontological STatistics) software^68^. Phenotypic frequency data of the 12 traits were analyzed using the Shannon–Weaver diversity index (*H*) given as:

1 $$H=-\sum _{i=1}^{k}{p}_{i}ln{p}_{i}$$

where k is the number of phenotypic classes for a trait and ${p}_{i}\,$is the proportion of the total number of entries (n) in the i th (i) class.

### DNA Extraction and PCR analysis

DNA was extracted from young leaves of 20-day-old plants following the mini-scale method^69^. Each PCR was carried out in a 20 μl reaction volume containing 1 μl of MgCl_2_ free 10 × PCR buffer with (NH_4_)_2_SO_4_, 1.2 μl of 25 mM of MgCl_2_, 0.2 μl of 10 mM of dNTPs, 0.2 μl of 5 U/μl Taq DNA polymerase, 0.5 μl of 10 μM forward and reverse primers, and 3 μl (10 ng) of DNA using a 96-well thermal cycler. An additional 10 μl of mineral oil was added in each well to prevent evaporation. Amplification was carried out using a G-storm PCR machine (Gene Technologies Ltd., England). Amplification conditions were one cycle at 94 °C for 5 minutes (initial denaturation) followed by 35 cycles at 94 °C for 1 minute (denaturation), 55 °C for 1 minute (annealing), 72 °C for 2 minutes (extension) with a final extension for 7 minutes at 72 °C at the end of 35 cycles. After mixing with the loading dye, PCR products were run through polyacrylamide gels. A 50 bp DNA ladder was used to determine the amplicon size. Three 4 μl PCR products were resolved by running gel in 1 × TBE buffer for 1.5 to 2.5 h depending upon the allele size at approximately 90 volts and 500 mA electricity. Gels were then stained with 1 μg/mL of ethidium bromide and documented using a Molecular Imager gel documentation unit (XR System, BIO-RAD, Korea).

### Spearman’s Coefficient of Rank Correlation

Quantitative phenotypic data for tested germplasm have been reported previously^7^. In this study, however, only the quantitative traits’ values were used for the Spearman^’^s coefficient calculation. Morphological and genetic distances among the genotypes were estimated, and ranking was done using Spearman’s coefficient with SSR analysis, 6328 = $\frac{n(n-1)}{2}$. Rank coefficients (${r}_{s}$) were further calculated by Spearman rank correlation test, in which data were collected as ranks or were ranked after observation on some other scale^70^. To measure and compare the association between two criteria of rankings, Spearman devised the following formula for estimating rank correlation (${r}_{s}$):

2 $${r}_{s}=1-\frac{6{\sum }^{}{d}_{i}^{2}}{n({n}^{2}-1)}$$

where n = number of observations, and ${d}_{i}$_=_ differences between the two ranks of each observation.

In this study, ranking involved D^2^ distances of phenotypic data and SSR analysis. Given that the number of pairs was large, estimated ${r}_{s}$ values were further tested for significance using the criterion

3 $$t=\,r\sqrt{\frac{n-2}{1-{r}^{2}}}$$

With $n-2\,$degrees of freedom^70^.

### Analysis of genotypic data model based approach

The DNA fragment of each germplasm was amplified using SSR markers and analyzed using different software. Molecular weight for each amplified DNA fragment was measured using Alpha Ease FC 4.0 software. We determined, were using Power Marker version 3.25, the summary statistics, which included (i) the number of alleles per locus, (ii) the frequency of major alleles, and the polymorphism information content (PIC) values^71^. An unrooted neighbor-joining tree from molecular data was constructed using MEGA^72,73^. Also, the allele frequency data from PowerMarker were used for analysis with NTSYS-pc version 2.1^74^, with the pair-wise genetic dissimilarity coefficients calculated using “Nei1983/CSChord1967” distance^64,75^. We calculated a similarity matrix in the Simqual subprogram using the Dice coefficient, which was followed by cluster analysis using the sequential, agglomerative, hierarchical and nested (SAHN) subprogram using the unweighted pair-groups with the arithmetic averages (UPGMA) clustering method as implemented in NTSYS-pc. Population structure for 113 germplasm was constructed using STRUCTURE version 2.3.4^76,77^. The number of clusters (K) we investigated ranged 1 to 15. The analysis used five replications for each K value. The model was used following admixture and correlated allele frequency with a 5000 burn period and a run length of 50000. The output of the analysis was collected using the STRUCTURE harvester and to identify three as the best K value based on the LnP(D) and Evano’s ΔK^78^. The principal components analysis (PCA) in a visualization technique that is commonly used in multivariate statistics, whereby the user can identify eigenvectors and amounts of variance and cumulatively explained variances per component^79,80^ was conducted using the SPSS (Version 16). In order to summarize the major variance patterns in the multi-locus dataset, an analysis of molecular variance (AMOVA) was performed using GenAlEx V6.5^81^.

## Electronic supplementary material

Supplementary Table
