# Supplementary material for: Variability Assessment of Aromatic Rice Germplasm by Pheno-Genomic traits and Population Structure Analysis
Source: Sci Rep. 2018 Jul 2;8:9911. doi: 10.1038/s41598-018-28001-z (PMC6028394; doi:10.1038/s41598-018-28001-z)
Supplement: Supplementary file 1 — Supplementary Table [file 41598_2018_28001_MOESM1_ESM.doc]

**Supplementary information**

**Variability Assessment of Aromatic Rice Germplasm by Pheno-Genomic traits and Population Structure Analysis**

M. Z. Islam^1*^, M. Khalequzzaman^1^, M. K. Bashar^2^, N. A. Ivy^3^, M. A. K. Mian^3^, B. R. Pittendrigh^4^, M. M. Haque^5^ and M. P. Ali^6*^

*^1^Genetic Resources and Seed Division, Bangladesh Rice Research Institute (BRRI), Gazipur 1701, Bangladesh*

*^2^CIAT, HarvestPlus, Banani, Dhaka 1213, Bangladesh*

*^3^Department Genetics and Plant Breeding, Bangabandhu Sheikh Mujibur Rahman Agricultural University (BSMRAU), Gazipur 1706, Bangladesh*

*^4^Department of Entomology, Michigan State University, East Lansing, MI*

*^5^Department of Agronomy, Bangabandhu Sheikh Mujibur Rahman Agricultural University (BSMRAU), Gazipur-1706, Bangladesh*

*^6^Entomolgy Division, Bangladesh Rice Research Institute (BRRI), Gazipur-1701, Bangladesh*

*Corresponding author email address: [panna_ali@yahoo.com](mailto:panna_ali@yahoo.com), zahid.grs@gmail.com

**Table S1. Ranking of phenotypic and genotypic based Nei (1983) distance values between germplasm through D^2^ and SSR analysis**

| **Sl. No.** | **Germplasm** | **Cumulative Nei (1983) distance ranking** | **D^2^ genetic distance ranking** | **Ranking difference** |
| --- | --- | --- | --- | --- |
| 1 | Sakor | 18 | 107 | -89 |
| 2 | Sagardana | 6 | 43 | -37 |
| 3 | *Nunia* | 14 | 63 | -49 |
| 4 | Chini Sagar (2) | 88 | 108 | -20 |
| 5 | Meny | 52 | 83 | -31 |
| 6 | Tilkapur | 73 | 113 | -40 |
| 7 | Binaphul | 44 | 100 | -56 |
| 8 | Kalobhog | 75 | 95 | -20 |
| 9 | Jabsiri | 70 | 106 | -36 |
| 10 | Kalgochi | 42 | 22 | 20 |
| 11 | Chinisakkor | 55 | 96 | -41 |
| 12 | Chini atob | 64 | 44 | 20 |
| 13 | Noyonmoni | 101 | 86 | 15 |
| 14 | Saubail | 38 | 67 | -29 |
| 15 | Chinniguri | 91 | 77 | 14 |
| 16 | Kolomala | 82 | 57 | 25 |
| 17 | Begunmala | 74 | 85 | -11 |
| 18 | Gopalbhog | 59 | 68 | -9 |
| 19 | Tulsimoni | 69 | 101 | -32 |
| 20 | Jirabuti | 54 | 99 | -45 |
| 21 | Khirshaboti | 112 | 69 | 43 |
| 22 | Rajbut | 27 | 24 | 3 |
| 23 | Soru kamina | 99 | 52 | 47 |
| 24 | Kamini soru | 40 | 58 | -18 |
| 25 | Doiarguru | 78 | 89 | -11 |
| 26 | Premful | 32 | 55 | -23 |
| 27 | Begun bitchi | 3 | 15 | -12 |
| 28 | Elai | 13 | 3 | 10 |
| 29 | Gua masuri | 106 | 102 | 4 |
| 30 | Luina | 80 | 75 | 5 |
| 31 | Lal Soru | 72 | 35 | 37 |
| 32 | Chini Kanai | 48 | 94 | -46 |
| 33 | Kalijira (SG) | 68 | 97 | -29 |
| 34 | Rajbhog | 35 | 98 | -63 |
| 35 | Phili.kata.bhog | 77 | 110 | -33 |
| 36 | Baoibhog | 97 | 109 | -12 |
| 37 | Baoi jhaki | 23 | 111 | -88 |
| 38 | Jirabhog(B) | 37 | 72 | -35 |
| 39 | Chinigura | 28 | 93 | -65 |
| 40 | Tulsimala | 24 | 82 | -58 |
| 41 | Bashmati 370 | 30 | 17 | 13 |
| 42 | Uknimodhu | 71 | 104 | -33 |
| 43 | Ranisalut | 29 | 9 | 20 |
| 44 | Jira dhan | 66 | 105 | -39 |
| 45 | Gandhakusturi | 33 | 11 | 22 |
| 46 | Sakkor khora | 46 | 61 | -15 |
| 47 | Badshabhog | 103 | 103 | 0 |
| 48 | jirakatari | 62 | 39 | 23 |
| 49 | Desi katari | 113 | 29 | 84 |
| 50 | Thakurbhog | 83 | 33 | 50 |
| 51 | Tulsimaloty | 104 | 27 | 77 |
| 52 | Raduni pagal | 45 | 88 | -43 |
| 53 | Sugandhi dhan | 2 | 47 | -45 |
| 54 | Kalijira (LG) | 102 | 90 | 12 |
| 55 | Jesso balam | 47 | 84 | -37 |
| 56 | Dakshahi | 16 | 40 | -24 |
| 57 | Hatishail | 93 | 76 | 17 |
|  |  |  |  |  |
| **Sl. No.** | **Gerplasm** | **Cumulative Nei (1983) Distance Ranking** | **D^2^ Genetic Distance Ranking** | **Ranking Difference** |
| 58 | Khasa | 51 | 92 | -41 |
| 59 | Buchi | 34 | 25 | 9 |
| 60 | AwnedTAPL-545 | 84 | 20 | 64 |
| 61 | BlackTAPL-554 | 20 | 30 | -10 |
| 62 | StrawTAPL-500 | 1 | 19 | -18 |
| 63 | Dubsail | 21 | 79 | -58 |
| 64 | Duksail | 60 | 73 | -13 |
| 65 | Khaskani | 110 | 62 | 48 |
| 66 | Khazar | 9 | 2 | 7 |
| 67 | Basmati S.106 | 11 | 31 | -20 |
| 68 | BR5 | 63 | 91 | -28 |
| 69 | BRRI dhan34 | 92 | 53 | 39 |
| 70 | BRRI dhan37 | 36 | 54 | -18 |
| 71 | BRRI dhan38 | 56 | 59 | -3 |
| 72 | BRRI dhan50 | 5 | 16 | -11 |
| 73 | Khasa M.pura | 105 | 28 | 77 |
| 74 | Uknimodhu | 49 | 41 | 8 |
| 75 | Bawaibhog-2 | 31 | 18 | 13 |
| 76 | Chiniatob-2 | 87 | 56 | 31 |
| 77 | Tilokkachair | 81 | 65 | 16 |
| 78 | Begunbitchi-2 | 79 | 48 | 31 |
| 79 | Chinairri | 76 | 78 | -2 |
| 80 | Bhatir cikon | 65 | 70 | -5 |
| 81 | Gordoi | 53 | 36 | 17 |
| 82 | Dolagocha | 10 | 64 | -54 |
| 83 | Kalnania | 17 | 50 | -33 |
| 84 | Dhan chikon | 67 | 60 | 7 |
| 85 | Badshabhog-2 | 25 | 37 | -12 |
| 86 | Thakurbhog-2 | 12 | 21 | -9 |
| 87 | Khutichikon(1) | 108 | 34 | 74 |
| 88 | Sunduri samba | 57 | 112 | -55 |
| 89 | Basmati | 89 | 71 | 18 |
| 90 | Basmati 37 | 15 | 13 | 2 |
| 91 | Basnatu S. 187 | 19 | 7 | 12 |
| 92 | Tulsimala-2 | 94 | 49 | 45 |
| 93 | Chinisail | 98 | 32 | 66 |
| 94 | Malshira | 111 | 80 | 31 |
| 95 | Sadagura | 86 | 42 | 44 |
| 96 | Modhumadab | 8 | 66 | -58 |
| 97 | Parbatjira | 26 | 51 | -25 |
| 98 | Chinikanai | 109 | 46 | 63 |
| 99 | Meedhan | 107 | 87 | 20 |
| 100 | Gobindhabhog | 95 | 74 | 21 |
| 101 | Kataribhog | 61 | 45 | 16 |
| 102 | Fulkari | 39 | 81 | -42 |
| 103 | BU dhan2R | 7 | 12 | -5 |
| 104 | Padmabhog | 22 | 38 | -16 |
| 105 | Dudsail | 100 | 5 | 95 |
| 106 | Sakkorkhana | 58 | 6 | 52 |
| 107 | Maloti | 85 | 14 | 71 |
| 108 | Bashful | 4 | 4 | 0 |
| 109 | Kalijira T.64 | 41 | 10 | 31 |
| 110 | OvalT.-2990 | 50 | 23 | 27 |
| 111 | KalijiraT.-68 | 90 | 8 | 82 |
| 112 | KalijiraT.-74 | 96 | 26 | 70 |
| 113 | Kalobakri | 43 | 1 | 42 |

∑di^2^= 174132, n = 6328 r_s =_ 0.276, t = 3.41 (Significant).

Table S2. Pair wise population differentiation according to groups of populations as measured

by FST using GenAlEx 6.503 software.

| Population | P1 | P2 | P3 |
| --- | --- | --- | --- |
| P1 | 0.00 |  |  |
| P2 | 0.023* | 0.00 |  |
| P3 | 0.068* | 0.060* | 0.00 |

*Signiﬁcance at P < 0.05 at 1,000 permutations

**Table S3. List of 52 SSR markers used in this study**

| **Name of marker** | **Chro.No.** | **Position (cM)** | **Product size (bp)** | **Forward primer sequence (5 to 3)** | **Reverse primer sequence (5 to 3)** |
| --- | --- | --- | --- | --- | --- |
| RM5 | 1 | 94.9 | 113 | TGCAACTTCTAGCTGCTCGA | GCATCCGATCTTGATGGG |
| RM495 | 1 | 2.8 | 159 | AATCCAAGGTGCAGAGATGG | CAACGATGACGAACACAACC |
| RM431 | 1 | 178.3 | 251 | TCCTGCGAACTGAAGAGTTG | AGAGCAAAACCCTGGTTCAC |
| RM237 | 1 | 115.2 | 130 | CAAATCCCGACTGCTGTCC | TGGGAAGAGAGCACTACAGC |
| RM312 | 1 | 71.6 | 97 | GTATGCATATTTGATAAGAG | AAGTCACCGAGTTTACCTTC |
| RM283 | 1 | 31.4 | 151 | GTCTACATGTACCCTTGTTGGG | CGGCATGAGAGTCTGTGATG |
| RM452 | 2 | 58.4 | 209 | CTGATCGAGAGCGTTAAGGG | GGGATCAAACCACGTTTCTG |
| RM6 | 2 | 154.7 | 163 | GTCCCCTCCACCCAATTC | TCGTCTACTGTTGGCTGCAC Bottom of Form |
| RM322 | 2 | 49.7 | 112 | CAAGCGAAAATCCCAGCAG | GATGAAACTGGCATTGCCTG |
| RM489 | 3 | 29.2 | 271 | ACTTGAGACGATCGGACACC | TCACCCATGGATGTTGTCAG |
| RM338 | 3 | 108.4 | 183 | CACAGGAGCAGGAGAAGAGC | GGCAAACCGATCACTCAGTC |
| OSR13 | 3 | 53.1 | 0 | CATTTGTGCGTCACGGAGTA | AGCCACAGCGCCCATCTCTC |
| RM514 | 3 | 216.4 | 259 | AGATTGATCTCCCATTCCCC | CACGAGCATATTACTAGTGG |
| RM307 | 4 | 0 | 174 | GTACTACCGACCTACCGTTCAC | CTGCTATGCATGAACTGCTC |
| RM537 | 4 | 8.5 | 236 | CCGTCCCTCTCTCTCCTTTC | ACAGGGAAACCATCCTCCTC |
| RM551 | 4 | 8.5 | 192 | AGCCCAGACTAGCATGATTG | GAAGGCGAGAAGGATCACAG |
| RM178 | 5 | 118.8 | 117 | TCGCGTGAAAGATAAGCGGCGC | GATCACCGTTCCCTCCGCCTGC |
| RM413 | 5 | 26.7 | 79 | GGCGATTCTTGGATGAAGAG | TCCCCACCAATCTTGTCTTC |
| RM510 | 6 | 20.8 | 122 | AACCGGATTAGTTTCTCGCC | TGAGGACGACGAGCAGATTC |
| RM454 | 6 | 99.3 | 268 | CTCAAGCTTAGCTGCTGCTG | GTGATCAGTGCACCATAGCG |
| RM170 | 6 | 2.2-7.4 | 121 | TCGCGCTTCTTCCTCGTCGACG | CCCGCTTGCAGAGGAAGCAGCC |
| RM190 | 6 | 7.4 | 124 | GCATTGTCATGTCGAAGCC | CTAGCAGGAACTCCTTTCAGG |
| RM253 | 6 | 37 | 141 | TCCTTCAAGAGTGCAAAACC | GCATTGTCATGTCGAAGCC |
| RM314 | 6 | 33.6 | 118 | CTAGCAGGAACTCCTTTCAGG | AACATTCCACACACACACGC |
| RM455 | 7 | 65.7 | 131 | AACAACCCACCACCTGTCTC | AGAAGGAAAAGGGCTCGATC |
| RM118 | 7 | 96.9 | 156 | CCAATCGGAGCCACCGGAGAGC | CACATCCTCCAGCGACGCCGAG |
| RM125 | 7 | 24.8 | 146 | ATCAGCAGCCATGGCAGCGACC | AGGGGATCATGTGCCGAAGGCC |
| RM10 | 7 | 63.5 | 159 | TTGTCAAGAGGAGGCATCG | CAGAATGGGAAATGGGTCC |
| RM408 | 8 | 0-1.1 | 156 | CAACGAGCTAACTTCCGTCC | ACTGCTACTTGGGTAGCTGACC |
| RM25 | 8 | 52.2 | 146 | GGAAAGAATGATCTTTTCATGG | CTACCATCAAAACCAATGTTC |
| RM44 | 8 | 60.9 | 99 | ACGGGCAATCCGAACAACC | TCGGGAAAACCTACCCTACC |
| RM284 | 8 | 83.7 | 141 | TCCTTGTGAAATCTGGTCCC | GTAGCCTAGCATGGTGCATG |
| RM447 | 8 | 124.6 | 111 | CCCTTGTGCTGTCTCCTCTC | ACGGGCTTCTTCTCCTTCTC |
| RM223 | 8 | 80.5 | 165 | GAGTGAGCTTGGGCTGAAAC | GAAGGCAAGTCTTGGCACTG |
| RM342 | 8 | 78.4 | 141 | CCATCCTCCTACTTCAATGAAG | ACTATGCAGTGGTGTCACCC |
| RM515 | 8 | 80.5 | 211 | TAGGACGACCAAAGGGTGAG | TGGCCTGCTCTCTCTCTCTC |
| RM316 | 9 | 1.8 | 192 | CTAGTTGGGCATACGATGGC | ACGCTTATATGTTACGTCAAC |
| RM215 | 9 | 99.4 | 148 | CAAAATGGAGCAGCAAGAGC | TGAGCACCTCCTTCTCTGTAG |
| RM271 | 10 | 59.4 | 101 | TCAGATCTACAATTCCATCC | TCGGTGAGACCTAGAGAGCC |
| RM287 | 11 | 68.6 | 118 | TTCCCTGTTAAGAGAGAAATC | GTGTATTTGGTGAAAGCAAC |
| RM536 | 11 | 55.1 | 243 | TCTCTCCTCTTGTTTGGCTC | ACACACCAACACGACCACAC |
| RM144 | 11 | 123.2 | 237 | TGCCCTGGCGCAAATTTGATCC | GCTAGAGGAGATCAGATGGTAGTGCATG |
| RM19 | 12 | 20.9 | 226 | CAAAAACAGAGCAGATGAC | CTCAAGATGGACGCCAAGA |
| RM20 | 12 | 0 | 144 | ATCTTGTCCCTGCAGGTCAT | GAAACAGAGGCACATTTCATTG |
| RM277 | 12 | 57.2 | 124 | CGGTCAAATCATCACCTGAC | CAAGGCTTGCAAGGGAAG |

Monomorphic markers

| RM124 | 4 | 150.1 | 271 | ATCGTCTGCGTTGCGGCTGCTG | CATGGATCACCGAGCTCCCCCC |
| --- | --- | --- | --- | --- | --- |
| RM507 | 5 | 0 | 258 | CTTAAGCTCCAGCCGAAATG | CTCACCCTCATCATCGCC |
| RM433 | 8 | 116.0 | 224 | TGCGCTGAACTAAACACAGC | AGACAAACCTGGCCATTCAC |
| RM105 | 9 | 32.1 | 134 | GTCGTCGACCCATCGGAGCCAC | TGGTCGAGGTGGGGATCGGGTC |
| RM474 | 10 | 0 | 252 | AAGATGTACGGGTGGCATTC | TATGAGCTGGTGAGCAATG |
| RM484 | 10 | 97.3 | 299 | TCTCCCTCCTCACCATTGTC | TGCTGCCCTCTCTCTCTCTC |
| RM552 | 11 | 40.6 | 195 | CGCAGTTGTGGATTTCAGTG | TGCTCAACGTTTGACTGTCC |
